# Learning in Plants: Lessons from *Mimosa pudica*

**DOI:** 10.3389/fpsyg.2016.00417

**Published:** 2016-03-31

**Authors:** Charles I. Abramson, Ana M. Chicas-Mosier

**Affiliations:** ^1^Department of Psychology, Laboratory of Comparative Psychology and Behavioral Biology, Oklahoma State UniversityStillwater, OK, USA; ^2^Department of Integrative Biology, Laboratory of Comparative Psychology and Behavioral Biology, Oklahoma State UniversityStillwater, OK, USA

**Keywords:** plants, learning, classical conditioning, instrumental conditioning, operant conditioning

## Abstract

This article provides an overview of the early *Mimosa pudica* literature; much of which is in journals not easily accessible to the reader. In contrast to the contemporary plant learning literature which is conducted primarily by plant biologists, this early literature was conducted by comparative psychologists whose goal was to search for the generality of learning phenomena such as habituation, and classical conditioning using experimental designs based on animal conditioning studies. In addition to reviewing the early literature, we hope to encourage collaborations between plant biologists and comparative psychologists by familiarizing the reader with issues in the study of learning faced by those working with animals. These issues include no consistent definition of learning phenomena and an overreliance on the use of cognition. We suggested that greater collaborative efforts be made between plant biologists and comparative psychologists if the study of plant learning is to be fully intergraded into the mainstream behavior theory.

## Introduction

The purpose of this article is threefold. First, it will provide a brief review of the early plant learning literature with a focus on *Mimosa pudica*. Much of *Mimosa* research was performed in the 1960s and early 1970s and is therefore rarely used for present scientific reference. This research appeared in psychological journals that are no longer easily accessible. As but one example in a recent book describing the behavior and intelligence of plants, no mention is made of the contributions of such comparative psychologists as Applewhite, Armus, Holmes, and Levy (Trewavas, 2014).

Second, we will provide an overview of non-associative and associative learning and the necessary control procedures from the perspective of psychology. We believe that this is especially important because we hope that this article stimulates collaboration between plant biologists and comparative psychologists. The study of plant learning was begun by comparative psychologists interested in the search for generalized learning phenomena but this interest appeared to have been short-lived. Despite this decline in interest, comparative psychologists still have much to offer the plant biologist with respect to the study of learning and intelligence. For example, comparative psychologists can contribute to philosophical discussions related to the terms cognition, intelligence, and learning, can contribute to novel approaches to data analysis, can design behavioral experiments, construct automated apparatus, and have much to say about levels of learning. In a recent article by Affifi (2013) levels of learning are discussed without any reference to the work of such comparative psychologists as Maier and Schneirla (1964) and Razran (1971). Moreover, in regards to learning paradigms, we do not say that in the course of conducting plant experiments no novel research designs will be developed – many have. However, these novel research designs can only be properly understood by reference to traditional behavioral designs created by psychologists. We may not be voicing the popular opinion but we would predict that few plant biologists interested in the study of learning have ever taken an undergraduate or graduate course on learning.

The plant biologist on the other hand can clearly contribute to comparative psychology by broadening our perspectives on learning and intelligence, forcing us to re-evaluate our assumptions on what is behavior, and introducing a new generation of comparative psychology students to the exciting developments in plant science and methods used to investigate plant behavior and physiology. We know of no comparative psychologist who has ever taken a course in plant biology – clearly we need to if we are going to have fruitful interactions with our colleagues in plant science. The potential benefits of plant biologists and comparative psychologists engaging in collaborative research is, in our view, untapped and can lead to some potentially exciting and important results. Third, this article will discuss some of the pitfalls and solutions that befall researchers when conducting learning research especially in regards to plants.

Why are there so few data driven studies on learning in plants and *Mimosa* specifically? In contrast to data driven studies we have found many papers concerned with justifying plant learning and intelligence (Trewavas, 2003, 2009; Cvrčková et al., 2009; Affifi, 2013; Debono, 2013b; Guiguet, 2013; Marder, 2013). For example, Trewavas (2003, 2009) and Cvrčková et al. (2009) discuss future directions and definitions of plant learning but do not present data. Similarly, Affifi (2013), Guiguet (2013), and Marder (2013) offer aspects of plant development that could be used to study learning but have not completed the necessary studies. Meyer et al. (2014) suggests that seed abortion can be considered a learned behavior, however, spontaneous abortion and stress induced abortion are common and may not be a complex decision as implied (Zhang et al., 2011).

In addition to articles focusing on what might be called philosophical issues, there is a lack of observational data on the behavior of plants. This is a necessary first step in the design of learning experiments. Observational research allows the researcher to establish baseline activity and response levels of the subject (Barnett, 1963). This baseline provides clues into methods of reward, aversive, and discriminative stimuli that can be incorporated into learning paradigms. Once baseline observational data are established, identification of motivating stimuli is required to develop a suitable standardized methodology that can then be compared to other behavior studies.

If a researcher is interested in studying operant conditioning, for example, a reward must be found that can be administered quickly, does not produce rapid satiation, and is effective over several presentations. Many learning paradigms require an established sequence of behavior that requires the delivery of time sensitive feedback. For example, one of the basic issues in operant conditioning is how to reward a behavior that does not naturally occur. One strategy is to reward successive approximations of the target response. This process, known as shaping, requires a reward to be administered at a precise time for producing a small piece of the desired action. Over time, the successive approximations come together to produce the final desired outcome. For this type of training, plants present unique challenges because they often appear to be inactive, making small behavioral changes difficult to see and relate to consequence. Lastly, to do a plant learning experiment correctly, environmental factors must be controlled. One method is to conduct the experiments in a growth or environmental chamber which are relatively expensive and must be modified to incorporate the apparatus necessary to control the behavioral aspects of the experiment.

## History of Learning in *Mimosa pudica*

Perhaps the first study of learning in plants was a habituation experiment reported by Pfeffer (1873) using the sensitive plant *Mimosa*. In this experiment, repeated mechanical stimulation of leaflets led to a decrease in sensitivity. Bose (1906) also looked at habituation of leaf closing in *Mimosa*. Bose confirmed Pfeffer’s findings and extended them by reporting that electrical stimulation, in addition to mechanical stimulation, can initiate leaflet closure. He also showed that a sufficient rest period was necessary before leaflet closure could be evoked again. It is important to note that he used an automated apparatus during this experiment.

Following Bose’s findings, the question naturally arises as to whether *Mimosa* can discriminate between stimuli. This was answered in the affirmative by Holmes and Gruenberg (1965) whose experimental design included a drop of water and finger touch as stimuli. After the plant habituated to water drops, the leaflet was touched with a finger. Finger touch now elicited leaflet closure even though the leaflet no longer responded to a drop of water. This experiment is important for two reasons. First, it provides data that the habituation of leaflet closure were not the result of fatigue. If the results were due to fatigue the leaflets would not respond to finger touch. Secondly, the results suggest that *Mimosa* can discriminate between stimuli.

The results of Holmes and Gruenberg (1965) motivated Applewhite (1972) to investigate whether some of the training variables known to influence habituation in animals also influence the habituation of *Mimosa*. In his experiment, Applewhite (1972) varied interstimulus interval (the time between stimulus presentations) and showed that as the interstimulus interval increased so did the time needed for habituation. Applewhite’s experiment was unique in at least two aspects: he used a preparation where leaflets were detached from the stem and placed in water (as opposed to using leaflets attached to the stem as others have done) and, he employed a dishabituation control. Unfortunately, given the previous results of Holmes and Gruenberg (1965) on the ability of *Mimosa* to discriminate between stimuli, he was not able to demonstrate dishabituation.

The study by Holmes and Gruenberg (1965) may be the first formal investigation of classical conditioning in plants. The purpose of this study was to condition *Mimosa* by pairing a light touch to a leaflet as the conditioned stimulus (CS) with an electric shock unconditioned stimulus (US). The electric shock was administered at the base of the plant. After 4 days of stimulus pairings the experiment showed no evidence of conditioned responses. The inability to find classical conditioning in *Mimosa* was confirmed by Holmes and Yost (1966).

Successful classical conditioning in *Mimosa* was, however, reported by Haney (as cited in Applewhite, 1975). In this procedure a change in illumination was the CS and touch was the US. While the experiment reported some conditioned responses to the CS, the experiment is difficult to interpret because of a lack of control groups (Applewhite, 1975). In further research, Armus (1970) replicated the experiment using a similar design and included a backward conditioning control group. However, attempts by Levy et al. (1970) failed to replicate the results of the original Haney experiments thereby calling into question the replicability of Armus (1970).

Additionally, Thomas performed an experiment on classical conditioning in *Mimosa* that took advantage of the finding that, under field conditions, the leaflets of *Mimosa* slowly drop as dusk approaches and slowly rise at dawn (personal communication). In the experiment, the CS was turning on the light in the growth chamber and the US was touching selected leaflets. Thomas found that leaflets in the paired condition showed conditioning compared to a light only or alternating stimulation condition. This finding should be replicated with controls for pseudoconditioning.

The most comprehensive and recent study of habituation of *Mimosa* was performed by Gagliano et al. (2014). Using leaflet closure as the dependent variable, and vertical dropping of the plants as the stimulus, the results confirm Holmes and Gruenberg (1965). Gagliano et al. (2014) employed a more controlled technique and investigated more phenomena including short and long term recall and the effect of light intensity. Both Holmes and Gruenberg (1965) and Gagliano et al. (2014) contained a control for dishabituation. Given the importance of the Gagliano et al. (2014) experiment and the failure to replicate some previous experimental results it is critical that the Gagliano et al. (2014) results be repeated by an independent laboratory.

In addition to *Mimosa*, habituation has been found in the carnivorous plant *Drosera* (sundew). When sundew tentacles are repeatedly stimulated they stop curling toward the stimulus (Pfeffer, 1906). In addition to sundew, Applewhite (1975) cites an experiment by Darwin reportedly showing habituation in the passion flower (*Passiflora gracilis*). During this experiment, when Darwin mechanically stimulated the passion flower tendrils, the tendrils no longer responded after 54 h of training. Using a different approach, Abramson et al. (2002) investigated the use of bioelectrical potentials as a method to explore plant behavior. Gold surface electrodes were placed on the upper surface of individual *Philodendron cordatum* with additional reference electrodes placed underneath. The dependent variable was the frequency of electrical activity detected by the electrodes. Plants were exposed to 6 h of light only, dark only, or alternating 1 min periods of light/dark. Following 6 h of “training,” all plants were exposed to a 10 min test period in darkness. The results revealed differences among the groups, but these differences were not interpreted as reflecting learning. However, the study did support the idea of using bioelectrical potentials with plants. We attempted to use the same procedure with *Mimosa*, but we could not reliably implant electrodes into the leaflets and stem. Debono (2013a) has also suggested that evoked extracellular activity at the level of the whole plant might be used as a dependent variable to investigate learning in plants. All these experiments need to be replicated with control procedures with individual data provided.

In an interesting article, Karpinski and Szechynska-Hebda (2010) discuss the intellect of plants from memory to intelligence. By studying recall, the researcher investigates a host of independent variables and that are solidly anchored to a set of dependent variables. This study focused on recall at the cellular level rather than as an externally observable behavior. The discussion of plant learning at various levels, from cellular to organismal and from different scientific fields is exactly what is advocated for in this article

## Types of Learning

In the 1960s, the psychological study of plant learning centered on the possibility of learning without a nervous system (Holmes and Gruenberg, 1965). There is also interest from behavioral scientists seeking to determine whether the similarities and differences in learning found among invertebrates and vertebrates could also be found in plants (Warden et al., 1940; Applewhite, 1975; Abramson et al., 2002; Guiguet, 2013).

The majority of early plant studies used the Sensitive plant (*M. pudica*). *Mimosa* has much to recommend it for learning studies. They are easy to maintain, much is known about its natural history, and they have a visible leaf closure response to external stimuli. However, there are drawbacks in the use of *Mimosa*, for example, it takes about 15 min for a leaf to recover (Holmes and Gruenberg, 1965) and not much is known about its genome in contrast to model species such as *Arabidopsis thaliana* whose entire genome is known.

The long recovery time is problematic because several training variables known to influence learning (such as the time between stimulus presentations, known as the interstimulus interval) and the time between a response and its consequence must be very short if an association is to be formed. This may also present a problem when comparing animal and plant behavioral techniques and studies because of the response time differential between organisms. Action potentials of animals take milliseconds to occur whereas similarly activated leaf closure in *Mimosa* may take seconds (Allen, 1969). Another limitation is the lack of automated conditioning procedures. Researchers must develop techniques for automatic presentation of stimuli and the automated recording of responses if the study of learning in plants is to reach the level of vertebrate, and some invertebrate, studies.

In the following section we will focus on methods to develop studies utilizing habituation, sensitization, and classical conditioning techniques. Instrumental and operant conditioning will not be covered because at this time there are no *Mimosa* studies in these areas; although one can envision a situation where the opening and closing of a leaf can be detected electronically. Once detected, the response would produce a consequence such as an airpuff or changes in light intensity. Detour experiments, in which animals are trained to go around barriers to reach some goal, could also be extended to plants (Kilgour, 1981; Wynne and Leguet, 2004). For example, a barrier can be placed in such a way that the growth of shoots or roots are blocked. Growth rate and direction of growth can be monitored to determine if the shoots or roots change their growth pattern. If so, the barrier can be removed and any change in behavior observed.

## Habituation

Habituation and sensitization are the most common paradigms for the study of non-associative learning. Habituation refers to a decrease in responding to a stimulus that is repeatedly presented. In order for this reduction to be considered an instance of learning, we must rule out sensory adaptation and motor fatigue. In general, most researchers recognize two types of habituation: short-term and long-term with the principal difference being the length of recall.

Studies of habituation show that it has several characteristics, including the following (Thompson and Spencer, 1966; Rankin et al., 2009):

(1) The more rapid the rate of stimulation is, the faster the habituation is.
(2) The weaker the stimulus is, the faster the habituation is.
(3) Habituation to one stimulus will produce habituation to similar stimuli (generalization).
(4) Withholding the stimulus for a long period of time will lead to the recovery of the response (spontaneous recovery).
(5) Habituation is a negative exponential function of the number of stimulus presentations.
(6) The rate of habituation increases as the number of training sessions increases.
(7) Presentation of a strong novel stimulus results in the return of the habituated response (dishabituation).
(8) Continued application of a dishabituation stimulus results in habituation of dishabituation.

## Sensitization

Sensitization, another category of non-associative learning, can be considered the opposite of habituation since it refers to an increase in the frequency or probability of a response, and can be divided into two categories: long-term and short-term. Studies of sensitization show that it has several characteristics including the following:

(1) The stronger the stimulus is, the greater the probability that sensitization will be produced.
(2) Sensitization to one stimulus will produce sensitization to similar stimuli.
(3) Repeated presentations of the sensitizing stimulus tend to diminish its effect.

Habituation and sensitization are well suited for the study of plant learning, since these behavioral phenomena are ubiquitous throughout the animal kingdom thereby providing an excellent database in which to compare and contrast results with plants. Furthermore, habituation and sensitization experiments are easy to perform – requiring little equipment with relatively simple experimental designs. Last, and perhaps most important, habituation and sensitization share many properties with more complex learning which creates unique opportunities to study behavior in plants. These properties, such as the ability of the response to recover over time; creating new behavior patterns; improvement in performance over successive sessions; and sensitivity to such training parameters as intensity, frequency, and pattern of stimulation can easily be investigated in plants. The importance of habituation and sensitization should not be underestimated. Perhaps not as exciting as demonstrating that a plant can manipulate a lever, non-associative learning is a fundamental behavior change and may be the only type of behavior modification found in plants.

Before a decrease in responsiveness can be attributed to habituation, several alternative explanations must be ruled out. The two most important are effector fatigue and sensory adaptation. In sensory adaptation, the decrease in responsiveness is associated with changes in sensory organs subjected to intense periods of stimulation. To rule this out you can select an intertrial interval – the time between presentations of the stimulus to be habituated – that is long enough to allow the effect of adaptation to subside. If long intertrial intervals are not practical, a test trial procedure can be substituted in which habituation is assessed not during training, but during test trials administered sometime after training. It is important to select a time interval between training and testing that is long enough for adaptation to dissipate. The available data on *Mimosa* does not contain studies investigating a wide range of intertrial intervals. Until such data are available and correlated with underlying physiological data, it is difficult to separate sensory adaptation from habituation.

Effector fatigue is a second source of error. This refers to the inability of the effector mechanisms responsible for the expression of the response to function properly. To separate the effects of fatigue from habituation, it is common to give the subject a test trial(s) using a second stimulus that also elicits the target response. If there is a response to this other stimulus (and there should be), and then a response to the reintroduction of the original training stimulus, the effect of fatigue may be ruled out. This procedure is known as dishabituation and is probably the most widely used control to assess the influence of fatigue in habituation experiments.

Before conducting and accurately interpreting the results of any habituation or sensitization experiment, it is important to know the rate, duration, and temporal pattern of the response that is to be conditioned. To establish a base rate of responding, add a control group to the experimental design and ensure that it is placed in the training situation but not given any habituation or sensitization training. Record the data as you would for a training run.

## Classical Conditioning

Classical conditioning is an example of associative learning and is generally thought to represent the most basic of the associative learning mechanisms (Razran, 1971). In classical conditioning a signal known as the CS is paired with a stimulus that elicits a reflex known as the US. After a number of CS-US pairings, the response elicited by the US (known as the unconditioned response) is now elicited by the CS (known as the conditioned response).

There are two major classes of classical conditioning experiment based on the type of US: if the US is something positive such as food it is known as appetitive conditioning and if the US is something aversive such as shock, it is known as aversive or defensive conditioning. Studies of classical conditioning show that it has several characteristics, including the following:

(1) In general, the more intense the CS is (up to a point) the greater the effectiveness of the training.
(2) In general, the more intense the US is (up to a point), the greater the effectiveness of the training.
(3) In general, the shorter the interval is between the CS and the US, the greater the effectiveness of the training.
(4) In general, the more pairings there are of the CS and the US, the greater the effectiveness of the training.
(5) When the US no longer follows the CS, the conditioned response gradually becomes weaker over time and eventually stops occurring.
(6) When a conditioned response has been established to a particular CS, stimuli similar to the CS may elicit the response.

Before it can be concluded that the appearance of a conditioned response is the result of the formation of an association between the CS and US, we need to eliminate alternative explanations including pseudoconditioning. Under this condition, an US is presented over the course of several trials and then a CS is introduced, often resulting in a response resembling that elicited by the US. This response is not considered a learned response because it was not the result of stimulus pairings. In order to estimate the amount of pseudoconditioning, the experimenter may use two control procedures. This decision is based on whether the researcher plans to use a group or single subject design. In a between group design, the control group will receive the same number of conditioned stimuli and unconditioned stimuli as the experimental group, but the stimuli are separated by an intertrial interval. Conditioning would be demonstrated by between group differences in the number and pattern of conditioned responses. In the case of a within group design using plants, the plant serves as its own control and would be trained to discriminate between two stimuli. The stimulus paired with an US is known as the CS+ and the second stimulus (not paired with the US) is known as the CS-. Classical conditioning would be demonstrated if the plant is able to discriminate between them in regards to the number and pattern of conditioned responses.

Pseudoconditioning is an interesting phenomena in its own right but seldom studied (i.e., Wickens and Wickens, 1942; Harris, 1943; Razran, 1971) and is certainly a phenomenon that can be studied in plants such as *Mimosa* (Holliday and Hirsch, 1986). In pseudoconditioning experiments a repetitive stimulus such as touch would be administered to a plant for a period then a secondary stimulus such as a shock would be administered to the same area of the plant. If the organism has a reduced response to the secondary stimulus then pseudoconditioning has occurred (Terry and Hirsch, 1997). Another way of conceptualizing this is that instead of associating a CS and US, a situation is arranged where two USs are associated.

One paradigm that deserves special consideration is known as alpha conditioning (Razran, 1971), in which the CS is not neutral. In other words, the CS already elicits a response that resembles the conditioned response. For example, with *Mimosa*, a very light touch can serve as the alpha CS followed by an US consisting of a strong touch which elicits leaflet closure. After a number of light touch/hard touch pairings, the leaflets may fold in response to the light touch. Such a paradigm can be easily automated and can lead to many interesting experiments. Alpha conditioning is seldom studied and Gormezano et al. (1983) do not mention the paradigm in their discussions of classical conditioning. As there are no generally accepted taxonomies of learning paradigms it is difficult for behavioral scientists to determine where this paradigm fits in. For example, Razran (1971) has argued persuasively that alpha conditioning is actually a form of instrumental behavior (i.e., behavior controlled by its consequences) and not classical conditioning. In our example of the *Mimosa*, a light touch is presented to a leaflet eliciting partial closure. This is closely followed by a tactile stimulus that elicits full leaflet closure. Over a number of pairings of slight touch and a strong tactile stimulus the leaflet may begin to fully close in response to the light touch. In this case the rewarding stimulus would be some protective or defensive response that now is generalized to the light touch. Clearly, alpha conditioning should be tried with *Mimosa* and other plants. The procedure is easy to implement and an associative effect may result as long as the study uses proper control groups. In this case, the study requires a single stimulus (an US presented at two different strengths) as opposed to the two needed in classical conditioning (CS and US).

## Presentation of Potential Methodologies for Learning in Plants

For adapting classical conditioning procedures to the study of plants such as *Mimosa* we would strongly recommend reading Gormezano and Kehoe (1975), Gormezano et al. (1983), and Gormezano (1984). These articles demonstrate two classical conditioning paradigms that could be utilized in plants including, Conditioned Stimulus-Conditioned Response (CS-CR), and Conditioned Stimulus-Instrumental response (CS-IR). These articles also demonstrate that there is no consistent definition of classical conditioning in the vertebrate (and invertebrate) learning literature.

The CS-CR paradigm represents the most basic case of classical conditioning. Here the experimenter has direct control over the relevant training variables such as stimulus intensity, number of training trials, interstimulus interval (time between stimulus presentations), and intertrial interval (time between trials). Furthermore, the CS does not elicit the unconditioned response prior to training and the conditioned response comes from the same effector system as the unconditioned response. Consider a hypothetical experiment with the sensitive plant, *Mimosa*. In this example, the CS would be flash of light and the US would be touch to a leaflet. Initially, the increase in illumination does not elicit leaflet closure but over the course of training the light will elicit closure.

The CS-IR paradigm contains designs known as transfer of control or classical-instrumental transfer. Unlike the above paradigm which measures classical conditioning directly, the CS-IR paradigms measures classical conditioning indirectly – by the ability of the CS to influence ongoing behavior. In the vertebrate literature the best known example is conditioned suppression in which a CS, such as light or sound, is paired with an US such as electric shock. After several stimulus pairings, the CS is presented during some type of ongoing behavior such as a lever press. The number of presses immediately prior to the introduction is the baseline measurement and is compared to the number of lever presses emitted during the presentation of the CS (no shock is presented). If a classical conditioning association was formed between light and shock, the presentation of the light would reduce the number of lever presses when compared to the time immediately before the introduction of the light. We have used conditioned suppression to assess the effect of insect repellents on honey bees (*Apis mellifera;* Abramson et al., 2006, 2010). In our *Mimosa* example, this design could be represented by pairing a stimulus such as a gentle touch or air-puff, with an aversive stimulus such as an electric shock or other intense stimuli. Data could be collected when the CS (touch) is presented while the plant is engaged in some behavior such as the opening of a leaflet, or turning toward a source of illumination (instrumental response). It would be expected that the movement response would be reduced in reaction to the touch which has previously been paired with an aversive stimulus.

## General Issues Related to the Study of Learning

There are many definitions of learning (Zimbardo, 1992). All definitions of learning contain several important principles. First, learning is extrapolated from behavior and is never observed directly. Rather, what we call learning is implied from observable and reproducible data. Second, learning excludes changes in behavior produced by, for example, development, fatigue, adaptation, or circadian rhythms. Third, temporary fluctuations are not considered learning. Rather, the change in behavior identified as learned must persist, if such behavior is appropriate. A fourth principle found in the definition is that some experience with a situation is required for learning to occur.

One way to address these definitional issues related to conditioning paradigms is to do away with such concepts as learning and intelligence. For example, Trewavas (2003, p. 1) mentions in the opening paragraph that “Intelligence is a term fraught with difficulties in definition.” This begs the question – why continue to use such terms? The concept of intelligence, for example, has been criticized by some psychologists as illogical, vague, and circular (Schlinger, 2003). Schlinger (2003) suggests replacing intelligence with a functional description of the contingencies and experimental conditions that produced the behavior. This view point has a long tradition in psychology beginning with the work of Schoenfeld (1966, 1972). Similarly, Markoš and Cvrěková (2013) point out that scientific words that become colloquial are not understood equally by every audience. Synonymous definitions require long-term usage by both the public and scientific audience that results in a single definition (Markoš and Cvrěková, 2013). Many words in psychology such as cognition and intelligence have not reached this point yet.

## Taxonomies of Learning

The work of Schlinger and Schoenfeld highlight the problems with definitions of learning such as classical conditioning, and more general terms such as intelligence. They suggest that one way to approach this issue is to create behavioral taxonomies. Both Bitterman (1962) and Tulving (1985) discuss how taxonomies can help researchers design and characterize learning experiments. Over the years several taxonomies have been proposed but none adapted. These include Bitterman (1962) for both classical and operant conditioning, Dyal and Corning (1973) and Gormezano and Kehoe (1975) for classical conditioning and Woods (1974) for instrumental and operant conditioning. In Woods (1974) classification he identifies 16 categories of conditioning based on the presence or absence of a discriminative stimulus and the desirability of the reward. The study of plant behavior offers a unique opportunity to revisit the behavioral taxonomy issue.

If a researcher is embarking on a research program investigating the behavior of plants, it seems reasonable to have a definition for plant behavior. Meyer et al. (2014) discuss “complex conditional decision making in plants” and point out that much of the evidence of behavioral plasticity in plants is based on physiological data with little contact with what social scientists would call behavior. One way Meyer et al. (2014) attempted to address this problem by using statistical models to understand seed abortion patterns in barberry plants exposed to environmental challenges. While this approach is fruitful and interesting, it does not demonstrate learning in an individual plant. When attempting to interpret plant learning in terms of cognitive constructs, it is important to recall that simple psychological answers should be assumed over complicated solutions (Morgan, 1898/1977).

Perhaps one way to get around the problem of a lack of taxonomies in learning research is to use mathematical models of the learning processes. These models can be applied to plant data and comparisons can made on the basis of, for example, differences in exponents (Stepanov and Abramson, 2008). Learning models have successfully been applied in a wide variety of situations including the effects of pesticides on learning in bees (De Stefano et al., 2014) and the assessment of recall in multiple sclerosis patients (Stepanov et al., 2012).

## The Reporting of Individual Data

Very few learning studies present examples of individual data with the exception of work in the area of behavioral analysis (Sidman, 1960). Most studies focusing on group data fail to reveal the shape of individual learning curves nor do they give information about the variation among plants or leaflets. This reliance on group data could lead to statements about species characteristics that are not reliable or valid and could lead to a misinterpretation (Hirsch and Holliday, 1988; Stepanov and Abramson, 2008; Grice, 2011; Grice et al., 2012; Craig et al., 2014). We would recommend that all studies of plant learning attempt to include individual data.

## Automation of Experiments

Plant learning is a relatively underdeveloped field of research; therefore there are no commercially available automated apparatuses for the study of plant learning. There are many companies that sell behavioral apparatus for both invertebrates and vertebrates but no company yet sells apparatuses to study the learning of plants. Therefore, any apparatus must be custom built. This problem is compounded by minimal construction skills of many faculty and students, limited access to constructing facilities, and little to no data that can be used to guide the design of an apparatus to study plant behavior. Further challenges include the high cost of behavioral control equipment and the required programing skills. The Propeller microcontroller (Parallax, Rocklin, CA, USA) and similar products offer a simple solution to regulate such behavioral experiments. These microcomputers are small, easily adapted to any environment or growth chamber, and inexpensive (<$100.00). The Propeller specifically, has a number of free programs written for fundamental conditioning paradigms such as habituation, classical conditioning, and operant conditioning (Varnon and Abramson, 2013).

## Recommendations

In closing the authors would like to make several recommendations. The most obvious is that greater attention be paid to investigating experimentally the possibility of learning in plants. Philosophical speculation is certainly interesting and forms an integral part of behavioral analysis but it cannot replace laboratory work. The work of Marder (2012, 2013) and Debono (2013b) on intentionality, attention, and cognitive perception is certainly thought provoking but is limited because the basic learning data are simply not available. Researchers should also become more familiar with the issues in the psychology of learning, particularly the comparative analysis of learning. A particularly important issue is the use of control groups (Abramson, 2013). As we have pointed out, it is difficult to get excited about research findings unless alternative explanations are ruled out. Studies of habituation, for example, must utilize a control for effector fatigue and sensory adaptation. The best way to do this is to incorporate a dishabituating stimulus as was done by Holmes and Gruenberg (1965), Applewhite (1972), and Gagliano et al. (2014). Moreover, studies of classical conditioning must employ a control group receiving the same number of stimuli but presented unpaired. The unpaired control group (or the use of a discrimination procedure when a within subject experimental design is employed) is necessary to assess the amount of pseudoconditioning. The use of a control group in which the response and consequence are unpaired is also necessary for studies of instrumental and operant conditioning. Additionally, including a “truly random” group with no contingency between conditioned and US may be used, (Rescorla, 1967). Alpha conditioning should also be investigated and the authors would also urge the use of mathematical models of learning and the use of behavioral taxonomies. This would help facilitate the comparison of learning procedures.

We would recommend that researchers create a catalog of stimuli that can serve as positive and negative reinforcers, punishments, conditioned, unconditioned, and discriminative stimuli. Before a learning experiment can be designed, researchers must know what will motivate a plant and for how long. The search for positive reinforcers is especially critical. In the absence of available positive reinforcers, aversive stimuli can be used but a comparative analysis of learning in plants cannot rest solely on the use of aversive events especially since aversive stimuli often damages plants.

The search for appropriate stimuli that can be used in learning experiments go hand in hand with the development of automated techniques that can be used to study training variables known to influence learning (i.e., interstimulus intervals, magnitude of reward). An effort must be made toward the development of automated training apparatus for plants. The authors also suggest that examples of individual data be reported in learning studies.

There needs to be an outlet for quantitative data in this field in order to help motivate the development of appropriate experimental equipment. The authors encourage journal editors and reviewers to support manuscripts that describe apparatus and report quantitative data related to the learning of plants. Especially important is the support of manuscripts that report negative results. The reporting of negative results will give researchers an idea of what worked and what did not, thereby saving valuable time.

We would also encourage psychologists, especially those interested in learning and comparative psychology, to interact with colleagues in botany, plant physiology, and philosophy. The possibility of higher order types of learning in plants is, in many ways, frightening and challenges the very foundation of learning theory and underlying physiological and biochemical mechanisms. Collaborations are a two way street and we have much to learn from one another. An interesting place to start is the work of Affifi (2013) on the possibility of levels of learning in plants. The notion of levels of learning has long been a basic tenet of comparative psychology (e.g., Warden et al., 1940; Razran, 1971).

## Conclusion

We believe the study of learning in plants is an exciting enterprise with the potential for creating valuable contributions in several areas of science. This article could be one of the first steps to encouraging scientists working on plants to embark on an experimentally based research program in which the psychology of learning and comparative psychology forms a central part.

## Author Contributions

CA wrote and provided the primary intellectual material for this manuscript. AC-M revised and acquired additional sources for the manuscript.

## Conflict of Interest Statement

The authors declare that the research was conducted in the absence of any commercial or financial relationships that could be construed as a potential conflict of interest.
